# The Impact of Reinforcement Ratio on the Punching Shear of CFRP Grid-Reinforced Concrete Two-Way Slabs

**DOI:** 10.3390/ma17225576

**Published:** 2024-11-15

**Authors:** Ning Duan, Jiwen Zhang

**Affiliations:** Civil Engineering School, Southeast University, Nanjing 211102, China; duan.ning@seu.edu.cn

**Keywords:** punching shear, CFRP grid, two-way concrete slab, reinforcement ratio

## Abstract

Corrosion of steel reinforcement in concrete slabs undermines structural durability and shortens the lifespan of concrete structures. Carbon Fiber-Reinforced Polymer (CFRP) is a promising material offering benefits such as high strength, corrosion resistance, and light weight. This study aims to investigate the punching shear performance of concrete slabs reinforced with CFRP grids, focusing on the effects of different reinforcement ratios. A series of experiments were conducted on two-way concrete slabs reinforced solely with CFRP grids to assess their punching shear resistance. Experimental results show that the CFRP grid achieves an ultimate tensile strength of 2181 MPa, with the cracking load of CFRP grid-reinforced slabs reaching approximately 20% of the ultimate load, highlighting a strong correlation between the ultimate load and grid reinforcement ratio. The observed punching failure exhibited clear brittle characteristics, characterized by the formation of radial and circumferential cracks on the tensile surface of the slab. The reinforcement ratio significantly influences the failure mode of the slabs; as the reinforcement ratio increases, the ultimate punching loads also increase. Additionally, a mathematical formula is proposed to predict the punching bearing capacity, achieving calculation errors below 20%. These findings contribute valuable insights into using CFRP grids as primary reinforcement, enhancing the design and application of durable concrete slab structures.

## 1. Introduction

Normally, cracks exist on the surface of concrete structure at service stage. External gases and liquids penetrate into the surface of the steel bars along the cracks, which will reduce the bonding synergy between the steel bars and the concrete. The corrosion and expansion of the steel bars will further expand the internal cracks in the concrete and evolution, ultimately leading to reduced durability of the concrete structure and premature failure of the structure [1,2]. From a material perspective, fiber composite material is a very promising material replace steel reinforcement in which corrosion reduces structural durability [3].

CFRP Grid is a fiber composite material with a new structure developed on the basis of carbon fiber reinforcement. It is mixed with carbon fiber and matrix material in a certain proportion and pressed with a special mold. The fibers are continuously distributed in two directions and have the same cross-sectional area, elastic modulus, tensile strength and other properties in the longitudinal and transverse directions. They also have the advantages of corrosion resistance, high strength, light weight and stable performance [4,5]. Its two-dimensional planar structure The form is naturally suitable for the repair and reinforcement of concrete slabs and wall structures.

The slab column structure, a prominent load bearing framework, comprises horizontal flat slabs and vertical columns, representing a prevalent structural paradigm in construction. This system offers several advantages, including straightforward design, economic feasibility, reduced building height, and streamlined construction processes [6,7]. However, a significant drawback lies in its vulnerability to central punching failure, a primary failure mode observed in reinforced concrete slabs subjected to concentrated loads, known as punching shear. This failure occurs when a plate slab experiences concentrated loads, such as heavy furniture items or point loads from columns [8,9].

The slab column structure represents a load-bearing framework comprising horizontal flat slab and vertical columns, constituting a prevalent structural paradigm in the realm of construction endeavors. This structural system offers advantages such as a straightforward design, economic feasibility, diminished building height, and streamlined construction processes. However, a notable drawback arises in its susceptibility to central punching failure. One of the primary failure modes observed in reinforced concrete slabs subjected to concentrated loads is known as punching shear. This type of failure occurs when a plate slab is subjected to a concentrated load, such as a heavy furniture item or a point load from a column.

Punching shear is characterized by a circular or elliptical failure surface around the point of maximum shear stress, resulting from a combination of shear stress and normal stress that leads to cracks at the slab perimeter and the formation of a cone shaped failure zone. During the service life of a structure, the deflection at the mid-span of a concrete slab under concentrated loading may exceed the permissible limit. When confronted with extreme loads, design deficiencies, or improper construction practices, the high concentration of shear stress at the column edges of concrete slab column structures under concentrated loading can precipitate brittle shear failure in the structure. Subsequent load redistribution could potentially lead to progressive collapse failure in the building structure [10,11,12].

The factors influencing punching shear failure in slabs can generally be categorized into three aspects: material properties, geometric characteristics, and loading conditions. Specifically, these include loading conditions, reinforcement ratio, slab thickness, support conditions, concrete strength, and the shape and size of the slab [13,14,15,16]. The influence of steel bars on concrete slabs is complex, involving various aspects such as the form of the steel bars (longitudinal reinforcement, transverse reinforcement), reinforcement ratio, yield strength of the steel bars, and pinning effect of the steel bars.

Scholars from around the world have conducted extensive experiments on punching shear failure and load-bearing capacity of concrete slabs. Alam conducted experiments on 15 slabs with boundary constraints and found that when the reinforcement ratio reaches 1.5%, the effect of the degree of bending reinforcement on the punching ultimate bearing capacity of the plate is minimal [17]. However, when the reinforcement ratio is low, the bearing capacity of the slab increases with an increase in the reinforcement ratio. Elstner (1956) and others proposed to consider the impact of reinforcement on the punching performance of concrete slabs from the reinforcement ratio of tensile reinforcement and the yield strength of reinforcement through the punching test of concrete two-way slabs [18]. Yitzhaki introduced the tensile-reinforcement strength ρ fy into the calculation formula for punching shear capacity, and believed that the bending failure and punching failure modes of slab column joints could be determined based on this parameter [19]. The relationship between the reinforcement ratio and punching shear capacity is due to the fact that increasing the reinforcement ratio of the flexural reinforcement reduces the depth and width of incline cracks, thus increasing the contribution of aggregate interlocking and the contribution of uncracked concrete to the punching shear capacity.

With the increase in the reinforcement ratio of concrete slab, the failure type of concrete slab will gradually transition from bending failure to bending and punching failure, until the pure punching failure of concrete slab occurs. The influence of the various literature on the reinforcement ratio is related to how to calculate the contribution of the reinforcement ratio. When the reinforcement ratio is low, the slab is more prone to punching shear failure due to its inability to resist concentrated loads. As the reinforcement ratio increases, the slab is better equipped to distribute the load and resist the formation of cracks, thus increasing its resistance to punching shear failure. However, the exact relationship between the reinforcement ratio and punching shear failure is complex and depends on several factors, including the type and arrangement of reinforcement, size and geometry of the slab, and the material properties of the concrete and reinforcement [20,21]. The effect of steel yield strength on punching shear capacity has a significant effect on member deformation.

Reinforcement is typically provided in the form of additional steel bars or, in some cases, fiber-reinforced polymer (FRP) grids to prevent punching shear failure. The objective of reinforcement is to increase the strength and stiffness of the slab and improve its resistance to punching shear. The use of fiber composite materials in engineering can be divided into two categories, such as reinforcing and repairing existing structures, and replacing ordinary steel bars with fiber grids in concrete structures [22,23]. FRP materials have advantages, such as light weight, high strength, magnetic resistance, and corrosion resistance. The integrity of FRP grid bars effectively inhibits crack expansion and evolution, reducing crack width. FRP materials have high specific strength, tensile properties, and good mechanical properties, making them a suitable replacement for steel reinforcement materials in structures [24].

The punching shear behavior of concrete slabs reinforced has been extensively studied in the literature [25,26]. However, there is limited exploration into their potential as a complete replacement for steel bars as the primary reinforcement in concrete slabs [27]. This study aims to assess the impact of CFRP grids on the punching shear performance of two-way concrete slabs, with a particular focus on their effectiveness as a substitute for steel reinforcement across varying reinforcement ratios. The innovation of this study is to evaluate the feasibility of completely replacing steel bars with CFRP grid. The study will provide a new idea for the selection of steel replacement materials by proposing and verifying the reinforcement effect of CFRP grid.

The medium and long term objectives of this study are to investigate the specific performance of CFRP grid-reinforced concrete structures in terms of load-bearing capacity, safety, and durability under various working conditions, thereby providing more practical parameters for their application in real engineering projects. In turn, this will enrich the knowledge base of civil engineering materials science and promote the use of new composite materials in the field of building structures.

## 2. Experimental Procedure

### 2.1. Specimen Design

Previous experimental studies on reinforced concrete slabs have indicated that the punching failure of such slabs is significantly influenced by the strength of the concrete, while the reinforcement ratio of reinforcing materials varies across different codes. Herein, CFRP grid reinforcement with equal strength in both vertical and horizontal directions is used to investigate the effect of the reinforcement ratio of CFRP grid reinforcement on punching shear failure. Three slabs (S1, S2, and S3) were designed, and the mechanical properties throughout the entire loading process were tested.

**Figure 1:**
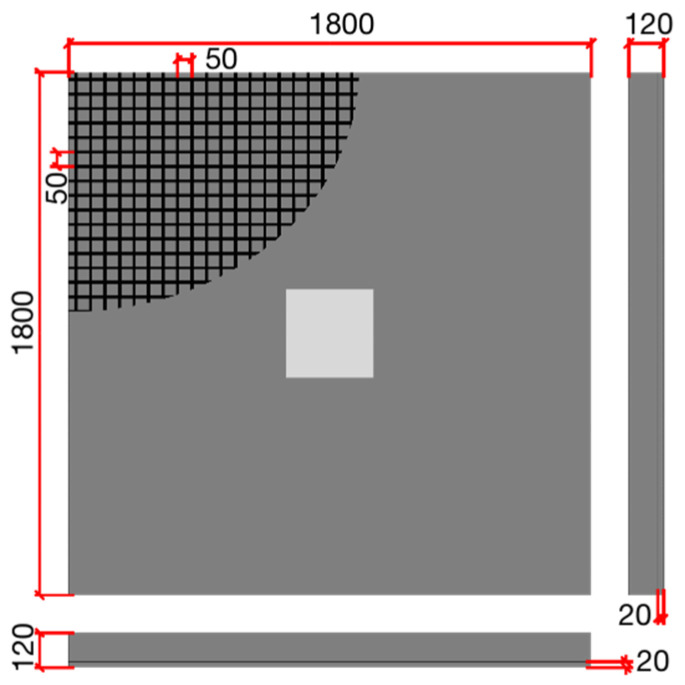
Schematic diagram of specimen.

Although the use of shear reinforcement can effectively enhance the punching performance of concrete slabs, this study employs only a single layer of CFRP grid on the tensile face of the slab to reduce the correlation between various influencing factors and achieve the objective of controlling experimental variables. Specifically, the CFRP grid is only placed on the tensile face of the concrete slab, without considering reinforcement on the compressive face or shear reinforcement. The detailed design parameters for different punching shear failure tests are shown in Table 1. The schematic diagram of the specimen is shown in Figure 1.

### 2.2. Materials

#### 2.2.1. Concrete

The mechanical performance experiments were conducted in accordance with the “Standard for Test Methods of Mechanical Properties of Ordinary Concrete” (GB50081-2002 [28]) at the civil engineering laboratory of Southeast University. To assess the compressive strength $f_{cu}$ of concrete, standard test blocks with dimension of 150 × 150 × 150 mm were cast during the pouring of concrete slab components. Subsequently, these test blocks underwent curing under the same conditions as the slab before undergoing punching failure tests. The concrete utilized in the specimens of this study is commercial concrete, with a design strength of C30. The cement employed is P.O. 42.5 ordinary Portland cement. The resultant effective test data derived from the measured cube compressive strength are averaged to determine the measured cube compressive strength $f_{cu}$.

The average compressive strength of the concrete is established at 23.1 MPa, with a recorded tensile strength of 2.6 MPa. The test outcomes for the compressive strength of concrete cubes, experimental test values, and the corresponding converted compressive strength and tensile strength are presented in Table 2.

#### 2.2.2. CFRP Grid

**Figure 2:**
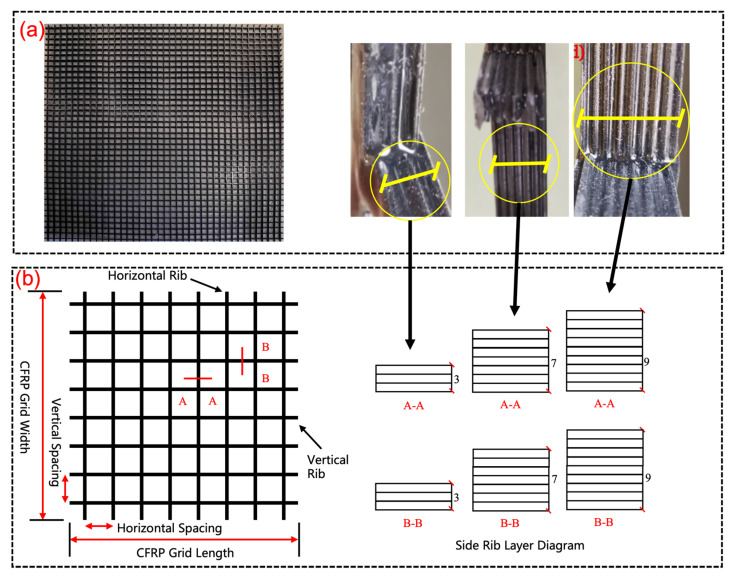
The CFRP grid: (**a**) The physical picture; (**b**) The diagram view.

Figure 2 illustrates the physical and diagram appearance of the CFRP grid used in the tests. The CFRP grid reinforcement comprises longitudinally and transversely aligned ribs of equal strength and layers, with rib dimensions of “3 + 3 @ 50”, “7 + 7 @ 50”, and “9 + 9 @ 50”. Here, “3 + 3”, “7 + 7”, and “9 + 9” denote the number of layers of ribs in the longitudinal and transverse directions, and “@50” refers to the rib spacing of 50 mm.

The CFRP grid reinforcement used in the study is a continuous product from Jiangsu Hengshen Co., Ltd. in No. 777 Tonggang Road, Danyang City, China. It was subsequently cut into 1750 × 1750 mm pieces, in line with the experiment’s requirements. Owing to the inherent characteristics of the production process, the cross-sectional dimensions of the composite grid are subject to discrete variations. Therefore, the effective area is established based on the nominal cross-sectional area value, and a single rib has a cross-sectional area of 6.67 mm^2^.

The tensile mechanical performance experiment of the CFRP grid bars is conducted at the Civil Engineering Laboratory of Southeast University. The loading is carried out using a hydraulic servo experimental machine, and the strain of the CFRP grid bars is measured by strain gauges affixed to its surface. The CFRP grid tendon’s tensile strength, elastic modulus, and elongation at break are determined via a tension test, which is performed using a displacement controlled loading method at a rate of 1 mm/min. The primary focus of the CFRP tendon tensile performance test is to evaluate the tensile strength, elastic modulus, and elongation at break. The CFRP tendon is found to be brittle in nature, and its fracture surface appears broom shaped.

The stress–strain curve of the CFRP tendon is observed to be essentially linear, without the presence of a distinct yield section. The material test results obtained for the tensile properties of the CFRP grid single leg tendon are presented in Table 3. The average ultimate tensile strength of the CFRP tendon is determined to be 2181 MPa, while the average elastic modulus is 123.6 GPa, and the average strain rate is 1.79.

### 2.3. Instrument

The primary test parameters for the CFRP grid-reinforced concrete slab punching test include the tensile and compressive strains on both the upper and lower surfaces of the concrete slab, the strain of the CFRP grid reinforcement, the deflection of the slab, and the applied load values.

**Figure 3:**
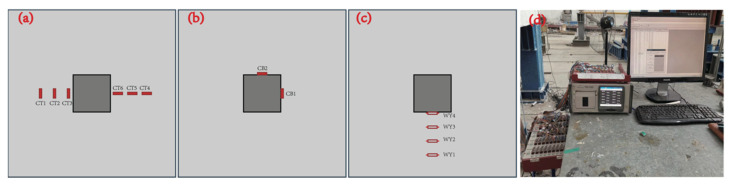
Measuring point and monitor devices of strain and displacement. (**a**,**b**) Concrete strain of slab top and bottom side. (**c**) Reinforcement Strain. (**d**) TDS 530.

The TDS530 static data acquisition instrument is used to collect the data of concrete strain, CFRP tendon strain, force sensor and displacement meter during the loading process, shown as Figure 3d. A TDS530 mainframe, a secondary computer and a computer mainframe were used in this test data collection. Use TDS 530 data acquisition software (Version 1.3.5) to record test data synchronously and in real time.

The CFRP grid and concrete strain gauges used are all resistance strain gauges, with the standard resistance value set to 120 Ω. The gauge length of the concrete strain gauge is 100 mm. The arrangement of the measurement points is shown in Figure 3. Along the centerline of the loading point, four displacement gauges with a range of 100 mm are arranged on one side of the loading point.

### 2.4. Test Methods

**Figure 4:**
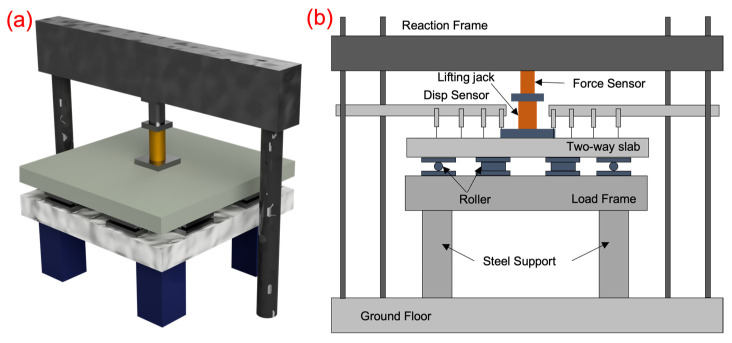
Tests loading device: (**a**) The 3D-loading diagram, (**b**) The main view.

Based on a comprehensive literature review, three loading methods have been identified for conducting punching failure tests. The first method involves applying a vertical concentrated load by arranging vertical supports, either by using short columns connected to the concrete slab or by directly placing load-distributing pads on the slab’s surface. In this setup, the central column of the slab is fixed, and a series of linear loads are applied around the perimeter of the slab. A less conventional approach involves positioning the slab vertically and applying lateral loads. After a thorough assessment, the method chosen for inducing punching shear failure in the concrete two-way slab consists of placing a steel pad at the center of the slab to apply a centrally concentrated load. The experimental setup for the punching shear test of the CFRP grid-reinforced concrete two-way slab is depicted in Figure 4.

During the test, a hydraulic jack is employed to apply the load, while the reaction force is provided by a reaction frame. The applied load is continuously measured throughout the process by a BLR tension compression force sensor positioned between the jack and the reaction frame. The experiment employs eight simply supported supports, evenly distributed on the loading frame, to restrict the vertical displacement of the concrete slab without hindering the free rotation of its edges. Each support measures 300 mm by 150 mm. The loading surface has dimensions of 300 mm by 300 mm, and the load is uniformly distributed using loading pads.

The testing procedure is divided into three stages: preparation, preloading, and formal testing. Prior to initiating the formal test, a preload of 20 kN is applied to the test specimen to ensure full contact between all components of the loading apparatus and the test piece. At this stage, the functionality of the specimen, the loading device, displacement gauges, and force sensors are carefully checked, and any issues are addressed promptly.

Once the formal test begins, a stepwise loading system is employed, with an increment of 20 kN at each step, continuing until the concrete slab undergoes punching failure. Each loading increment was maintained for a duration of 10 min, allowing for thorough observation and recording of the slab’s response until punching failure occurred. The experiment is load controlled throughout the punching failure process. During the test, the condition of the concrete slab is continuously monitored. When the sustained load is applied, crack development on the concrete slab is recorded to document the progression of the cracks. Given that the experiment proceeds in a forward direction, the tension surface of the slab is located on the underside, and only the applied load is recorded until it reaches 70% of the estimated load value at which crack development occurs.

## 3. Results and Discussion

### 3.1. The Load–Displacement

**Figure 5:**
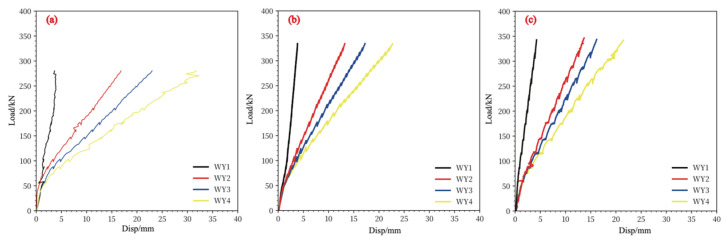
The load-displacement of slab with different reinforcement ratio. (**a**) 0.34%, (**b**) 0.78%, (**c**) 1%.

The P−δ curve on the central axis of the two-way concrete slab with CFRP grid is shown in Figure 5, the ultimate load and maximum displacement are listed in Table 4. The reinforcement ratio of CFRP grid has a significant effect on the punching shear capacity of concrete slabs. When the reinforcement ratio increased from 0.34% to 0.78%, the bearing capacity of the concrete slab increased significantly and the deflection decreased significantly. The load capacity has increased from 250 kN to around 350 kN and the deflection has decreased from 31 mm to around 22 mm. When the reinforcement ratio was increased from 0.78% to 1%, the bearing capacity and deflection of the concrete slab did not increase significantly.

During the loading period, record the development of cracks and record the load value when the cracks are first observed, and record the ultimate load value when the loading surface has scouring damage, $V_{cr}$ is the load value when the cracks are first observed, $V_{p}$ is the ultimate load value of the slab. The absolute value of the cracking load of the configured CFRP concrete slab are in the range of 40~80 kN, and the ultimate load values are between 250 kN and 350 kN. The cracking of the concrete slab has no obvious relationship with the CFRP grid bars, but is related to the properties of the concrete material itself; the punching load of the two-way concrete slab is related to the CFRP grid bars. With the increase in the cross-sectional area of the grid bars, the punching load of the concrete slab The carrying capacity is significantly increased.

In general, the P−δ curve of the CFRP grid reinforced concrete slab can be divided into two stages, which are represented by two straight lines with different slopes. The first stage is from the beginning of loading to the cracking load of the concrete slab, which is related to the initial stiffness of the specimen. The second stage is between the cracking load and the ultimate punching load. Due to the decrease in the stiffness of the concrete slab due to the generation of concrete incline cracks, the slope of the straight line in the second stage is smaller than that in the first stage.

### 3.2. Crack Patterns

#### 3.2.1. The Surface Crack

**Figure 6:**
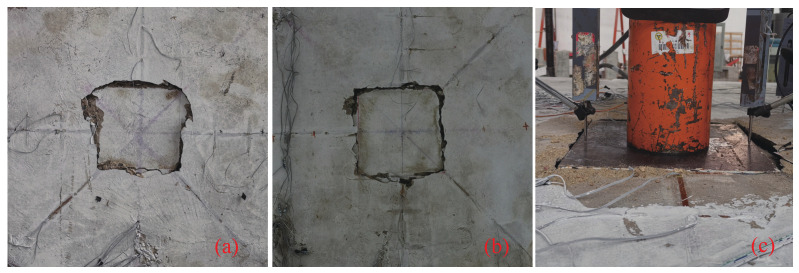
Distribution of cracks on the top surface of the slab: (**a**) S1, (**b**) S2, (**c**) S3.

**Figure 7:**
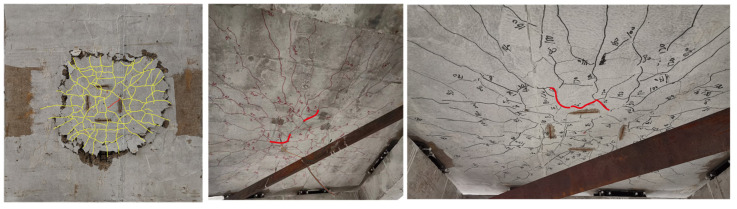
Distribution of cracks on the bottom surface of the slab. Red line indicated the first crack; yellow line indicated the distribution cracks after punching shear.

The morphological characteristics of the crack distribution on the top and bottom surface are illustrated in Figure 6 and Figure 7. In Figure 7, the red line indicated the first crack when loaded, and the yellow line in Figure 7 indicated the distribution cracks at the time of punching shear failure.

The crack distribution on the surface of specimen S2 is presented in Figure 6a. The compression surface of the concrete slab exhibits a ring of subsidence cracks surrounding the loading area, with no significant cracks observed elsewhere on the compression surface. On the left side of the loading area, some concrete crushing is evident. The tensile surface reveals distinct radial and circumferential cracks, which are fine in nature. At the junction between the punching cone and the concrete slab, there is noticeable concrete spalling, indicative of typical punching shear failure. The progression of crack development on the tensile surface is as follows: at a load of 80 kN, radial cracks begin to form at the bottom of the slab; at 100 kN, these radial cracks extend to the edge of the loading frame. Subsequently, circumferential cracks develop, with the first prominent closed circumferential crack appearing at a distance of 19–64 mm from the loading surface. Upon failure, a pronounced punching cone is formed, with the distance from the bottom of the cone to the loading surface ranging between 220 and 440 mm.

The crack distribution on the surface of specimen S2 is presented in Figure 6b. Similarly to specimen S1, the compression surface of the concrete slab shows a ring of subsidence cracks around the loading area, with no significant cracking observed in other regions. The crack development on the tensile surface proceeds as follows: at a load of 60 kN, the tensile surface begins to crack; by 120 kN, radial cracks at the bottom of the slab extend to the edge of the loading frame. The lower portion of the slab develops circumferential cracks. Unlike specimen S1, however, specimen S2 does not exhibit a punching cone at the bottom, nor is there any significant concrete spalling. Instead, a single ring of circumferential cracks forms at a distance of 80 mm from the edge of the loading surface.

The surface crack distribution characteristics of specimen S3 are shown in Figure 6c. It can be observed that only a ring of subsidence cracks formed around the loading area on the compression surface, with no significant cracks appearing elsewhere. After punching failure occurred, all loading pads were punched into the slab. The development of cracks on the tensile surface progressed as follows: at a load of 60 kN, radial cracks began to form on the tensile surface of the slab; at 100 kN, these cracks extended to the edge of the loading frame; at 120 kN, a circumferential crack was formed. Unlike specimen S1, specimen S3 exhibited failure characteristics similar to specimen S2, with no punching failure surface at the bottom of the slab and no evident concrete spalling.

The main difference between specimen S3 and specimens S1 and S2 is that specimen S3 developed an inclined crack on one side of the slab, which expanded towards the side edge of the slab during loading, positioned above the reinforcement cover layer. After the punching failure and subsequent loading, the cracks fully developed, dividing the slab into upper and lower parts along the grid reinforcement. As shown in Figure 7, a punching failure cone formed within specimen S3, but the inclined cracks did not penetrate the grid ribs nor reach the bottom of the slab. Instead, the cracks extended along the grid ribs to the slab’s side edge. After punching failure, continued loading resulted in the grid ribs at the bottom of the slab being loaded as a unified structure.

#### 3.2.2. The Incline Crack

**Figure 8:**
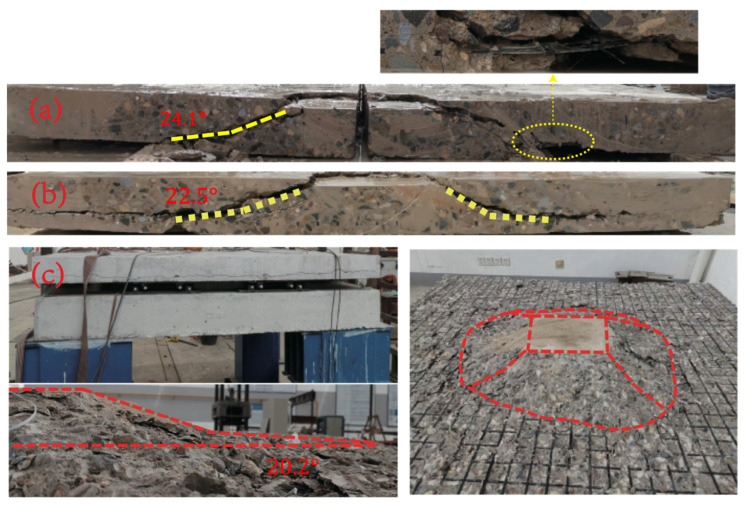
Distribution of the incline cracks, dash lines indicated crack boundary or location. (**a**) The specimen S1, (**b**) the specimen S2, (**c**) The specimen S3.

The analysis of the development process of slab ventral cracks during the loading process of the punching failure experiment is a challenging task due to the three dimensional characteristics of the concrete slab. In the existing research, there are two methods to observe the cracks of the slab. These methods include cutting along the central axis to observe the distribution characteristics of cracks and implanting strain measuring rods in the plate to analyze the changes in plate thickness during the experiment. The former is a relatively common technique for observing the shape of cracks in the slab. This study selects the method of cutting the concrete slab after the punching failure test to observe the distribution of incline cracks in the concrete slab. The incline cracks in the web of the slab are shown in Figure 8.

The cross-sectional view of the incline crack in specimen S1 shown as Figure 8a indicates that the incline cracks on the left side of the specimen have a “concave” shape, while the incline cracks on the right side are in the shape of incline straight lines. At the cutting section position, there are several incline cracks in the slab, one of which develops into a critical incline crack and runs through the upper and lower surfaces of the slab. The lower end of the critical incline crack is incline intersected with the grid reinforcement within 360 mm from the center of the slab, and the lower end of the critical incline crack is partially crushed. This aligns with the fact that a circle of concrete peeling occurs on the tensile surface of the concrete slab when the punching cone failure surface punches the slab body. The CFRP grid tendon at the junction of the critical incline crack and the grid tendon exhibits tearing phenomenon.

The cross-sectional view of the incline crack in specimen S2 shown as Figure 8b, on the other hand, reveals a “concave” distribution of the critical incline cracks. Multiple incline cracks are observed on the left side of the cross section diagram, one of which develops into a critical incline crack that penetrates the upper and lower surfaces of the slab, eventually expanding to the edge of the slab along the grid reinforcement during the final failure. The lower end of the critical incline crack is incline intersected with the grid reinforcement within 420 mm from the center of the plate, and partial concrete breakage occurs at the intersection of the incline crack and the grid reinforcement on the left. The excellent integrity of the CFRP grid reinforcement prevents all incline cracks in the slab from extending to the tension surface of the concrete slab, preventing the formation of penetrating annular cracks.

The cross-sectional view of the incline cracks of the specimen S3, as depicted in Figure 8c. It is noteworthy that the specimen S3 exhibits a distinct behavior compared to S1 and S2. Specifically, the incline cracks of S3 propagate towards the side edge of the plate during the loading process, which is situated above the protective layer. Upon reaching the punching loading stage, the plate is subjected to complete crack development, causing the plate to divide into upper and lower parts through cracks developed along the grid reinforcement. Notably, a punching failure cone is formed inside the specimen S3, and the incline cracks do not penetrate the grid ribs and extend to the bottom of the plate. Instead, they extend along the grid ribs to the side edge of the plate. Following the punching failure, loading is continued, and the grid ribs at the bottom of the slab are loaded as a single unit.

The characteristics of incline cracks in plate slab reinforced with CFRP grid are similar, and the incline cracks are “concave”, the crack extension ranges are between 300 and 400 mm, and the punching cone angle is 24.1°, 22.5° and 20.2°, the average inclination angle is 20.2°; it is far smaller than the value of the punching cone angle in the specification and the data measured in the punching test of the reinforced concrete slab.

### 3.3. Strain Distribution

#### 3.3.1. The Concrete Strain of Slab Tension Surface

**Figure 9:**
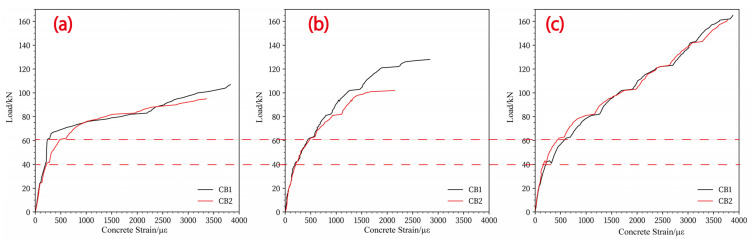
The concrete strain of slab tension face: (**a**) S1; (**b**) S2; (**c**) S3.

The force strain relationship of concrete on the tensile surface is presented in Figure 9. It can be observed that the strain trend of the concrete slab at the two measuring points adjacent to the loading surface follows a three stage pattern. In the first stage, the strain shows a linear growth trend, with the load value being around 40 kN and the strain approximately 200 με. In the second stage, the slope of the strain curve is lower than that of the first stage, and the load increases from 40 kN to 60 kN, resulting in a strain increase from 200 to 500 με. In the third stage, the strain grows rapidly as the load is further increased.

It has been observed that the load-carrying capacity of the concrete slab improves with an increase in the CFRP grid reinforcement ratio. At an applied load of approximately 60 kN, the concrete reaches an ultimate tensile strain of 1000 με, initiating crack formation, which subsequently propagates to the slab’s edges quickly. The tensile strain on the concrete surface at final failure is not significantly different, ranging from 3000 to 4000 με, with the concrete configured with 7 + 7 @ 50 exhibiting a relatively smaller tensile strain value. This may be due to the strain gauge ceasing to function early on account of the cracking of the concrete surface.

#### 3.3.2. The Concrete Strain of Slab Compression Surface

**Figure 10:**
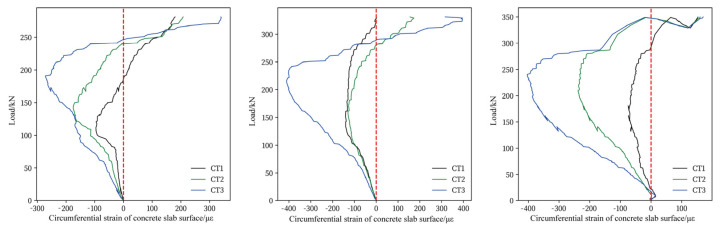
Circumferential strain of concrete slab compression surface: (**a**) S1, (**b**) S2, (**c**) S3.

**Figure 11:**
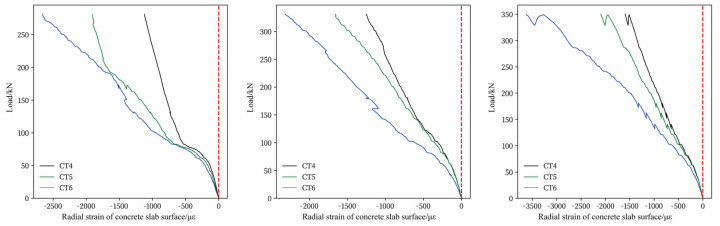
Radial strain of concrete slab compression surface: (**a**) S1, (**b**) S2, (**c**) S3.

Figure 10 and Figure 11 illustrate the circumferential and radial strain of the concrete slab’s compression surface under varying loads. The typical stress–strain curve reveals four distinct working stages for concrete, with the first stage being the elastic phase. As shown in Figure 10 and Figure 11, when the concrete strain is below 100 με, the concrete slab remains in the elastic phase. During this phase, the strain is directly proportional to the applied load, and deformation is minimal. As the applied load exceeds the elastic range, microcracks begin to form in the concrete. Figure 10 and Figure 11 indicate that when the concrete strain reaches approximately 300–400 με, the rate of strain growth significantly accelerates. Cracks on the slab’s tension surface develop rapidly, with severe cracks propagating toward the slab’s corners.

Comparing the circumferential and radial strains on the concrete surface in compression reveals distinct behaviors. As shown in Figure 10, the circumferential strain on the compression surface initially exhibits negative values, gradually increasing as the load rises. Upon reaching approximately 350 με, the circumferential strain increases with the applied load but subsequently begins to decrease. In the later stages of loading, the circumferential strain transitions to positive values, indicating that the concrete on the compression surface enters a tensile state. Figure 11 demonstrates that the radial strain of the concrete increases rapidly with the applied load, the strain value reaches its maximum value when the extreme load is reached. The compressive strain on the concrete surface decreases with increasing distance from the loading surface, and this variation is more prominent closer to the loading surface.

Furthermore, both circumferential and radial strain values on the concrete surface remain below 3300 με, the ulimate compression strain of concrete, indicating that punching shear failure exhibits a brittle failure mode. Notably, no cracks are observed on the compression surface of the slab prior to the punching shear crack formed.

## 4. Mathematical Model

The calculation of punching shear capacity for concrete slabs is a focal point for engineers worldwide when it comes to shear resistance design. To prevent shear failure in slab column structures, various countries’ codes have proposed corresponding design methods to enhance the ultimate load carrying capacity and deformation capability of joints, thereby avoiding or delaying brittle failure of these joints. The American Concrete Institute code is a widely referenced design standard for reinforced concrete structures. According to the ACI code, for square columns without shear reinforcement, the punching shear capacity P of concrete slabs is calculated using Equation (1). In accordance with ACI 318-19, the critical section of two-way members is defined to be located at a distance half of the effective slab thickness measured from the column faces.

(1) $$P_{aci}=0.33\lambda_{s}b_{0}d\sqrt{f_{c}^{\prime }}$$

where $\lambda_{s}$ is size effect modification factor, $b_{0}$ is the critical shear perimeter, d is effective thickness of slab (mm), and $f_{c}^{\prime }$ is unconfined compressive strength of concrete (MPa).

According to Equation (1), the punching shear capacity for the concrete slab reinforced with CFRP grid is calculated to be 254 kN. From the results, it appears that the calculations based on the ACI 318-19 tend to be conservative, applying this code for the design of CFRP reinforced concrete slabs leads to a structurally safe outcome. In Equation (1), the calculation of punching shear capacity does not consider the influence of reinforcement ratio, hence the calculated results do not reflect the impact of CFRP grid reinforcement. It is necessary to propose a punching shear capacity calculation method that takes into account the effect of reinforcement and conduct further analysis.

In 2005, Jacobson et al. conducted a test on the punching capacity of concrete slabs equipped with double layer glass fiber reinforced mesh elements, and compared the test results on the punching capacity with specifications such as ACI 318, ACI 400, Eurocode 2, and BS 8110 [29]. Based on the calculation results of Matthys mathematical model, a modified mathematical model for calculating the punching shear bearing capacity of FRP concrete slabs is proposed.

(2) $$P_{u,jacobason}=4.5\frac{\sqrt[{3}]{\rho f_{c}^{\prime }}}{\sqrt{d}}u_{1.5d}d$$

The form of grid bars adopted by Jacobason were different from the two-way equal strength grid bars used in this article, it is a grid form composed of smooth round carbon fiber bars connected by steel frame.

In 2004, El-Gamal et al. conducted a series of punching shear failure tests involving five concrete slabs equipped with GFRP bars and two concrete slabs equipped with CFRP bars, and proposed a calculation formula for the punching shear bearing capacity of FRP reinforced concrete slabs [30].

(3) $$P_{u,gamal}=0.33\sqrt{f_{c}^{\prime }}u_{0.5d}\alpha 1.2^{N}$$

(4) $$\alpha =0.5\sqrt[{3}]{\rho E_{f}}(1+\frac{8d}{u_{0.5d}})$$

Based on the above analysis and the punching shear failure test analysis results carried out in this article, the mathematical formula for calculating the punching shear bearing capacity of the CFRP grid reinforced concrete slab proposed in this article is as follows:

(5) $$P_{prop}=C_{1}C_{2}\frac{h_{z}}{d}f_{ct}u_{1.5d}d$$

where C1 is the size effect coefficient, C2 is a constant coefficient with a value of 0.55, $h_{z}$ is the height of the neutral axis, $u_{1.5d}$ is the calculated cross section The perimeter is taken as the most unfavorable perimeter of the vertical section of the plate at a distance of 1.5d from the periphery of the local load or concentrated reaction area.

(6) $$C_{1}=\frac{2}{\sqrt[{2}]{1+\frac{d}{200}}}{\left(\frac{d}{a}\right)}^{0.2}$$

(7) $$h_{z}=0.75d{\left(\frac{E_{frp}}{E_{c}}\rho \right)}^{\frac{1}{3}}$$

The calculation results of the above formulas are compared by calculating the error between the computed values and the experimental values. The formula for the error between the experimental values and the computed values is as follows:

(8) $$\epsilon =\frac{P_{calc}-P_{test}}{P_{test}}$$

Compare the calculation results of the above formula by calculating the error between the calculated value and the experimental value, show as Table 5. The calculation errors by proposed Equation (5) are 4%, 16% and 20%, respectively, which are better than the formula proposed by Jacobason and El-Gamal. Additionally, more low reinforcement CFRP grid reinforced concrete punching shear experiments are needed to extensively verify the applicability of the proposed formula.

## 5. Conclusions

This paper conducts an experimental study on the punching shear resistance of two-way concrete slabs configured with two-way equal strength CFRP grid reinforced concrete slabs. The main parameter of the experimental study is the reinforcement ratio of CFRP grid bars, which is represented by the different number of layers of grid bars. The research results show that the entire failure process of the concrete slab equipped with CFRP grid reinforcement has ductile failure characteristics, and the formation of the punching cone shows a brittle failure moment. When the reinforcement ratio of CFRP grid bars reaches a certain level, the concrete slab has better load-bearing capacity.

The tensile mechanical properties of CFRP grid were found to be excellent, with an average ultimate tensile strength of 2181 MPa, an average elastic modulus of 123.6 GPa, and an average elongation at break of 1.79%. These results demonstrate the potential of CFRP grid as a reinforcement material for concrete structures.

The ratio of the cracking load to the ultimate load of the CFRP grid-reinforced concrete slab was approximately 0.2. While the cracking load did not exhibit a significant correlation with the configuration of the grid bars, the ultimate load was found to be influenced by the reinforcement ratio of the grid bars. At a low reinforcement ratio of 0.33%, the deflection of the concrete slab was found to be large, with the position of the loading surface reaching 32 mm. However, as the reinforcement ratio increased to 0.78%, the deflection of the loading surface decreased to 23 mm. Moreover, further increases in the reinforcement ratio did not result in a significant increase in the deflection of the plate.

The punching failure behavior of the concrete slab was found to be characterized by brittle failure. Radial cracks appeared on the tensile surface of the slab at 40–80 kN, and multiple regions were divided by staggered radial and circumferential cracks at the ultimate load. The punching failure was marked by the formation of a ring of punching cracks around the loading surface, and the distribution of circumferential cracks on the tension surface of the plate was found to be in the range of 400–600 mm near the loading surface. These observations provide valuable information regarding the failure behavior of CFRP grid-reinforced concrete slabs. The average inclination of punching cone is 22.4°, smaller than the data measured in the punching test of the reinforced concrete slab.

The reinforcement ratio of CFRP grid reinforcement with equal longitudinal and horizontal strength was found to significantly affect the punching failure mode of the slab. For a reinforcement ratio of 0.33%, an obvious punching cone failure occurred, and the cone rushed out of the slab surface with a phenomenon of concrete spalling on the tensile surface. For reinforcement ratios exceeding 0.78%, the punching cone failure surface could not be formed on the tensile surface of the concrete slab. Moreover, when the reinforcement ratio was high, the incline cracks in the slab web developed along the grid reinforcement, and when the load value was high enough, the crack may extend to the side of the slab. These observations highlight the importance of optimizing the reinforcement ratio of CFRP grid reinforcement for improved performance of concrete slabs under punching shear.

Given the limited experimental data, next work will expand on these findings by employing finite element analysis (FEA) to gain a more comprehensive understanding of the behavior of CFRP grid-reinforced slabs under varied loading conditions. This approach will allow for deeper insights into the relationship between reinforcement ratio and slab performance, facilitating the development of more effective reinforcement strategies for concrete slab structures. Future investigations will include the performance of CFRP grids under various loading conditions, optimizing reinforcement ratios and configurations and examining the feasibility of CFRP grids for real-world applications, such as cost-effectiveness, ease of installation, and bonding performance with concrete.

## Figures and Tables

**Table 1:** Basic parameters of the test slab.

ID	Slab/mm	$h_{0}$/mm	CFRP Grid	Load Size/mm	ρ/%
S1-F1H12	1800 × 1800 × 120	100	3 + 3 @ 50	300 × 300	0.33
S2-F2H12	1800 × 1800 × 120	100	7 + 7 @ 50	300 × 300	0.78
S3-F3H12	1800 × 1800 × 120	100	9 + 9 @ 50	300 × 300	1

**Table 2:** The concrete strength.

ID	Dimension/mm	$f_{cu}$/MPa	$f_{c}$/MPa	$f_{t}$/MPa
1	150 × 150 × 150	31.2	23.7	2.6
2		30.2	23	2.6
3		29.4	22.3	2.5
4		30.7	23.3	2.6
Mean		30.4	23.1	2.6

**Table 3:** The performance of CFRP grid.

ID	$f_{u}$/MPa	E	e/%
1	1984	123.8	1.78
2	2277	122.5	1.84
3	2282	124.6	1.77
Mean	2181	123.6	1.79

$f_{u}$, The ultimate tensile strength. *E*, The elastic modules. *e*, The elongation at break.

**Table 4:** The ultimate load value and displacement of slab.

ID	ρ/%	P/kN	$d_{WY1}$/mm	$d_{WY2}$/mm	$d_{WY3}$/mm	$d_{WY4}$/mm
S1	0.33	281.3	4.0	17.0	23.2	32.3
S2	0.78	334.5	4.0	13.3	17.5	23.0
S3	1.0	350.4	4.3	13.5	16.2	21.7

**Table 5:** Error analysis of punching shear capacity calculation results.

ID	$\epsilon_{aci}/\%$	$\epsilon_{jacobason}/\%$	$\epsilon_{gamal}/\%$	$\epsilon_{prop}/\%$
S1	9	7	14	4
S2	10	19	12	16
S3	24	11	21	8

## Data Availability

Dataset available on request from the authors.
